# High Leucine Diets Stimulate Cerebral Branched-Chain Amino Acid Degradation and Modify Serotonin and Ketone Body Concentrations in a Pig Model

**DOI:** 10.1371/journal.pone.0150376

**Published:** 2016-03-01

**Authors:** Anna G. Wessels, Holger Kluge, Frank Hirche, Andreas Kiowski, Alexandra Schutkowski, Etienne Corrent, Jörg Bartelt, Bettina König, Gabriele I. Stangl

**Affiliations:** 1 Institute of Agricultural and Nutritional Sciences, Martin Luther University Halle-Wittenberg, 06120, Halle (Saale), Germany; 2 AJINOMOTO EUROLYSINE S.A.S., 75817, Paris Cedex 17, France; 3 Lohmann Animal Nutrition GmbH, 27472, Cuxhaven, Germany; The Nathan Kline Institute, UNITED STATES

## Abstract

In addition to its role as an essential protein component, leucine (Leu) displays several other metabolic functions such as activation of protein synthesis. This property makes it an interesting amino acid for the therapy of human muscle atrophy and for livestock production. However, Leu can stimulate its own degradation via the branched-chain keto acid dehydrogenase complex (BCKDH). To examine the response of several tissues to excessive Leu, pigs were fed diets containing two- (L2) and four-fold (L4) higher Leu contents than the recommended amount (control). We found that the L4 diet led to a pronounced increase in BCKDH activity in the brain (2.5-fold, *P* < 0.05), liver (1.8-fold, *P* < 0.05) and cardiac muscle (1.7-fold, *P* < 0.05), whereas we found no changes in enzyme activity in the pancreas, skeletal muscle, adipose tissue and intestinal mucosa. The L2 diet had only weak effects on BCKDH activity. Both high Leu diets reduced the concentrations of free valine and isoleucine in nearly all tissues. In the brain, high Leu diets modified the amount of tryptophan available: for serotonin synthesis. Compared to the controls, pigs treated with the high Leu diets consumed less food, showed increased plasma concentrations of 3-hydroxybutyrate and reduced levels of circulating serotonin. In conclusion, excessive Leu can stimulate BCKDH activity in several tissues, including the brain. Changes in cerebral tryptophan, along with the changes in amino acid-derived metabolites in the plasma may limit the use of high Leu diets to treat muscle atrophy or to increase muscle growth.

## Introduction

Leu is one of the branched-chain amino acids (BCAAs) and, in addition to playing a role as a substrate for protein synthesis, it has an important function in the stimulation of protein translation through the activation of *mammalian target of rapamycin* (mTOR) [1,2]. The nature of Leu as a nutrient signal that stimulates protein synthesis has encouraged scientists to study the efficacy of a high Leu supply as a strategy to prevent muscle wasting and cachexia in patients and to improve growth performance of livestock. Previous data have shown that high doses of Leu can promote muscle protein synthesis in neonatal pigs via activating factors downstream of mTOR [3,4]. In apparently healthy adults over 60 years of age, the administration of Leu-enriched whey protein supplements, compared to conventional dairy products, leads to a larger postprandial muscle protein synthesis [5]. Based on those findings, Leu has been considered as a therapeutic approach to attenuate skeletal muscle atrophy, which is frequently observed in patients suffering from trauma, sepsis and cancer [6,7].

However, in the liver, high levels of Leu can stimulate branched-chain keto acid dehydrogenase complex (BCKDH), an enzyme complex, that catalyzes the irreversible degradation of all BCAAs including valine (Val) and isoleucine (Ile) [8]. BCKDH consists of three subunits: the branched-chain α-keto acid decarboxylase (E1), a dihydrolipoyl transacylase (E2) and a dihydrolipoyl dehydrogenase (E3). BCKDH activity is regulated by the interconversion between the phosphorylated and dephosphorylated forms [9], and BCKDH catalyzes the degradation of BCAAs via decarboxylation of the transaminated branched-chain keto acids of Leu (α-ketoisocaproate), Val (α-ketoisovalerate) and Ile (α-keto-β-methylvalerate). In 1998, Suryawan and co-workers [10] reported marked species differences in tissue-specific BCKDH activity in humans, rats and African green monkeys. However, in pigs, one of the most important production animals and a potential human model, the effects of a high Leu diet on BCKDH activity have been investigated in only the liver and skeletal muscle [8,11].

In this study, we sought to elucidate BCKDH activity and the changes in the concentrations of free amino acids and amino acid derivates in several tissues, including the brain, in response to diets that contain excessive Leu. We used growing pigs as an animal model because, for pigs, in contrast to other species, the exact need for single amino acids has been well described, which enables an accurate formulation of their diets. Another important issue is that the anatomy and physiology of pigs closely resemble those of humans (reviewed in [12]). Like humans, pigs do not have a dietary requirement for protein per se, but they do have minimum dietary requirements for the same essential or amino acids as humans, along with an adequate amount of nonessential amino nitrogen from various nonessential amino acids (reviewed in [12]). These features are important not only in terms of developing possible therapies for humans but also with regard to the fact that intake of protein and Leu in industrialized countries is, on average, twice as high as the suggested need for this amino acid [13].

## Material and Methods

All experimental procedures described were approved by the council of Saxony-Anhalt (Landesverwaltungsamt Sachsen-Anhalt, Germany; approval number: H1-4/33A) and followed the general guidelines outlined in the European animal welfare regulations. Experiments were conducted in environmentally controlled facilities, and animals were housed in groups of two. During the experimental period of 35 days, the room temperature was incrementally reduced from 28°C to 25°C at the end of the study.

### Study design and diets

To investigate basal and Leu-stimulated BCKDH activity in pigs, 110 crossbred [Pietrain x (Large White x Landrace)] 35-day-old piglets of both sexes with an average initial body weight of 10.4 ± 0.7 kg (mean ± SD) were randomly assigned to three groups of 36–38 animals each. Piglets were blocked by sex, body weight and ancestry. The three groups of pigs received experimental diets that were based mainly on corn, wheat, barley and soybean meal as source of protein (S1 Table) and that differed in Leu supplementation. The analyzed Leu content of the control diet (10.9 g/kg, see S2 Table) matched the recently published Leu recommendation for piglets [14]. The high Leu diets were supplemented with additional amounts of L-Leu, mainly at the expense of glutamic acid, to achieve 200% (L2 group, analyzed diet content 19.7 g/kg) or 400% (L4 group, analyzed diet content 37.5 g/kg) of the Leu in the control diet. The other essential amino acids were added to the diets in quantities that met the requirements for essential amino acids [14] (S2 Table). The metabolizable energy content of the diets was 13.7 MJ/kg. The analyzed crude protein content of the diets was 15.5%. All diets were isonitrogenous and isoenergetic and were formulated to meet the requirements of minerals and vitamins [15]. The pigs were fed the experimental diets for 35 days.

Food and water were available *ad libitum*. The food intake and body weight of all pigs were recorded weekly. The gain:food ratio was calculated by determining the ratio between weight gain and food intake to elucidate possible effects of Leu on food conversion.

### Sample collection

On day 35 of the experiment, 10 pigs per group were captive-bolt stunned to take blood and tissue samples. To avoid differences in the amount of ingested amino acids before sampling, the pigs were food deprived for 12 h and then received an equal portion of food 2.5 h prior to sacrifice. Blood samples were centrifuged for 10 min at 3,000 g to obtain plasma for the analyses of amino acids, α-keto acids, 3-hydroybutyrate and serotonin. Samples from the pancreas, liver, kidney, longissimus dorsi muscle, cardiac muscle, brain (dorsal part the cerebrum), duodenal mucosa and adipose tissue (external fat of longissimus dorsi muscle) were collected to analyze BCKDH activity and to quantify free amino acids. To collect duodenal mucosa, a 10 cm part of the duodenum (starting 15 cm behind the pars pylorica) was excised, was washed several times with cold NaCl solution (0.9%) and was cut lengthwise. Intestinal mucosa was harvested by scraping the surface of the small intestine. The samples were snap frozen in liquid nitrogen and stored at -80°C until analysis.

### Nitrogen and amino acid quantification of the diets

The nitrogen (N) content of the diet was analyzed as described elsewhere [16]. The amino acid content of the diets was analyzed by a JLC-500/V AminoTac Amino Acid Analyzer (Jeol, Croissy-sur-Seine, France) in the laboratory of AJINOMOTO EUROLYSINE S.A.S. (Amiens, France) according to a previously described method [17]. To quantify methionine and cysteine, diet samples were oxidized with performic acid prior to hydrolyzation. Amino acids were separated by ion exchange chromatography and measured by photometric detection after derivatization with ninhydrin. Total tryptophan (Trp) was analyzed by HPLC after an alkaline hydrolysis with barium hydroxide [17].

### Analysis of BCKDH activity

The BCKDH activity assay was conducted with a spectrophotometer with α-ketoisovalerate as substrate, as described previously [18]. In contrast to the previous protocol [18], the extraction buffer was made with sodium fluoride instead of potassium fluoride, and the assay buffer was made without dihydrolipoamide dehydrogenase. Briefly, 150 mg tissue samples were homogenized in an extraction buffer (50 mM HEPES, 30% (w/v) Triton X-100, 2 mM EDTA, 5 mM sodium fluoride, 2% (v/v) bovine serum, 0.1 mM tosyl L-phenylalanyl chloromethyl ketone, 0.1 mg/ml trypsin inhibitor, 0.02 mg/ml leupeptin) with a Mixer Mill (MM 400, Retsch, Haan, Germany). After centrifugation at 20,000 g for 5 min at 4°C, the supernatant was incubated with 27% (w/v) polyethylene glycol. Following a second centrifugation step (13,000 g, 10 min, 4°C), the pellet was dissolved in a suspending buffer (25 mM HEPES, 0.1% (w/v) Triton X-100, 0.2 mM EDTA, 0.4 mM thiamine pyrophosphate, 1 mM DTT, 50 mM KCL, 0.02 mg/ml leupeptin and BCKDH activity was determined using α-ketoisovalerate as a substrate and by recording the NADH formation at 340 nm.

### Quantification of free amino acids from plasma and tissues

The concentrations of free amino acids in plasma and tissues were determined as isoindole derivatives by reversed phase HPLC (Hypersil ODS, 250 mm x 4 mm, 5 μm, Agilent 1100, Agilent Technologies, Waldbronn, Germany) [19] with fluorescence detection after pre-column derivatization with o-phthaldialdehyde and mercaptopropionic acid [20,21]. For quantification of free amino acids in tissues, the tissue samples were treated as described elsewhere [22]. Briefly, 150 mg of tissue was diluted in 0.6 mL 0.1 N hydrochloric acid, which contained 50 μM norvaline as the internal standard. After homogenization with a Mixer Mill (MM 400, Retsch, Haan, Germany), samples were centrifuged at 10,000 g for 15 min, and supernatants were diluted in acetonitrile 1:2. The protein-free supernatant was derivatized and used for HPLC analysis.

### Analysis of plasma and cerebral serotonin concentration

The concentration of serotonin in the plasma was analyzed by using the serotonin reagent kit (Chromsystems, Munich, Germany) for HPLC analysis (3030), the corresponding column (3130), the mobile phase (3031) and the electrochemical detector CLC 100 from Chromsystems Instruments & Chemicals GmbH (Munich, Germany). Sample analysis was performed according to the manufacturer’s protocol and with an Agilent 1100 HPLC (Agilent Technologies). To this end, 100 μL of plasma was mixed with 100 μL of the internal standard and 100 μL of the precipitation reagent for 30 seconds. The mixture was incubated for 10 min at 8°C, followed by centrifugation at 17,900 g for 10 min. A volume of 20 μL of the supernatant was injected into the HPLC system.

Cerebral samples for serotonin analysis were prepared as described previously [23]. Briefly, 10 mg of tissue samples was homogenized in 120 μL of ice-cold extraction solution (5 μM clorgyline containing 5 μg/mL glutathione and 20 ng/mL N-ω-methylserotonin as internal standard) using a Mixer Mill (MM 400, Retsch, Haan, Germany) for 1 min at 15 Hz and an ultrasonic bath RK 501 H (Bandelin electronic, Berlin, Germany) for 5 min at 0°C. For protein precipitation, 10 μL of 2 M HClO_4_ was added to the homogenate, followed by the addition of 8 μL 2.5 M potassium acetate. After mixing and incubation for 15 min on ice, the homogenate was centrifuged for 10 min at 15,000 g. Subsequently, 80 μL of the supernatant was diluted with 80 μL of the mobile phase. The HPLC analysis [24] was conducted with an Agilent 1100 HPLC with a Hypersil ODS column (250 mm x 4 mm, 5 μm, Agilent) at 30°C and the electrochemical detector CLC 100 of Chromsystems. The mobile phase contained 50 mM citric acid, 50 mM acetic acid, 11 mM decanesulfonic acid and 15% acetonitrile (v/v). The pH was adjusted to 4.5 with NaOH before the addition of acetonitrile. The flow was set to 1 mL/min and the injection volume was 50 μL.

### Quantification of plasma α-keto acids and 3-hydroxybutyrate

Concentrations of the plasma α-keto acids α-ketoisocaproate, α-ketoisovalerate and α-keto-β-methylvalerate were determined by HPLC after derivatization with o-phenylendiamine using α-ketocaproic acid as the internal standard [25]. Forty μL of plasma was mixed with 4 μL of 500 μM internal standard and 80 μL of 1 M HClO_4_. After a 10 min incubation at 4°C and centrifugation (10 min, 22,000 g, 4°C), 50 μL of the supernatant was mixed with 50 μL 25 mM o-phenylendiamine in 2 M HCl and incubated for 30 min at 50°C. After cooling to room temperature the samples were centrifuged again (5 min, 22,000 g, 4°C) and the supernatants were analyzed by HPLC with an Agilent 1100 with a Hypersil ODS column (250 mm x 4 mm, 5 μm, Agilent) at 30°C and fluorescence detection (350 nm/410 nm). The α-keto acid derivates were eluted by a gradient of methanol and water with a flow rate of 0.8 mL/min (0 min 32.5%, 5 min 32.5%, 10 min 41.5%, 12 min 55%, 20 min 88.5%, and 32 min 100% methanol). The injection volume was 10 μL.

Because Leu is a ketogenic amino acid, we hypothesized that a high Leu diet would increase plasma concentrations of ketone bodies. The plasma concentration of 3-hydroxybutyrate was analyzed with a test kit (Autokit 3-HB test system; Wako Chemicals GmbH, Neuss, Germany).

### Statistical Analyses

Statistical analyses were performed using SPSS Statistical Software (IBM SPSS Statistics Standard 20, Armonk, NY, USA). Data are presented as the mean ± standard deviation (SD) and were analyzed by General Linear Model ANOVA. Sex and ancestry were included in the statistical model as linear covariates for all performance parameters. Because sex and genetics had no effects on the results, they were excluded from the model. Values were analyzed for homoscedasticity by Levene’s test. The three groups were compared by Tukey’s test if variance means were homogeneous, and by Games-Howell test if variance means were unequal. Effects were considered to be significantly different at *P* < 0.05.

## Results

### High Leu diets led to a decline in food intake

**Fig 1:**
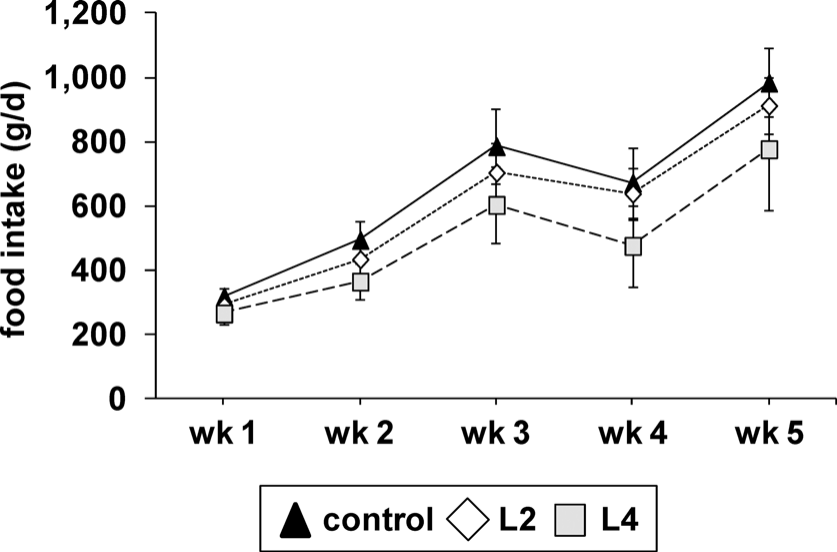
Average daily food intake of pigs fed diets with different Leu contents. Values represent the means ± SD of pigs that were fed a diet with the recommended amount of Leu (control) or with a two- (L2) or four-fold (L4) excess of Leu compared to the control diet for 35 days (average daily food intake of each pig was assessed by calculating the mean average value of 18–19 pens with two pigs per pen, n = 18–19). Food intake of was recorded weekly (wk 1 to wk 5).

In general piglets in all groups appeared to be healthy and did not show any obvious changes in behavior. Piglets that received the L2 and L4 diets showed a significantly lower daily food intake than the control piglets (Fig 1). The analysis of the average daily food intake over the entire experimental period revealed that the L2 group had 9% and the L4 group had 23% lower food intake than the control group (control, 638 ± 71 g/d; L2, 583 ± 45 g/d; L4, 490 ± 77 g/d; *P* < 0.001). The reduced food intake resulted in a reduction of growth as assessed by the weight gain of these pigs (control, 411 ± 87 g/d; L2, 379 ± 71 g/d; L4, 322 ± 80 g/d; *P* < 0.001). The gain:food ratio was not altered in response to the high Leu diets (control, 0.64 ± 0.02 kg/kg; L2, 0.64 ± 0.03 kg/kg; L4, 0.64 ± 0.06 kg/kg).

### Excessive Leu intake stimulates BCKDH activity in most tissues, including the brain

**Fig 2:**
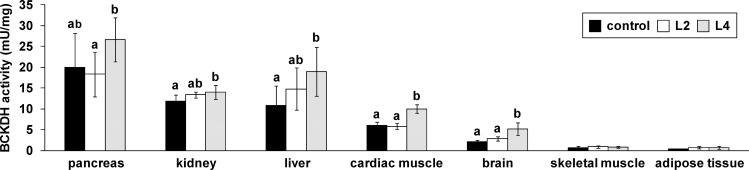
Branched chain keto acid dehydrogenase complex (BCKDH) activity in different tissues of pigs in response to diets with different Leu contents. Values represent the means ± SD of pigs fed a diet with recommended Leu content (control) or with two- (L2) or four-fold (L4) excess of Leu compared to the control diet for 35 days (n = 10). Data were analyzed by one-way ANOVA. Individual means of treatment groups per tissue were compared by Tukey’s test if variances were homogeneous. If variances were heterogeneous, as revealed by Levene’s test, individual means were compared by Games Howell test. ^a, b^Means not sharing a common letter are significantly different from one another (*P* < 0.05).

There were considerable tissue-specific differences in basal (control group) and stimulated BCKDH activity (Fig 2). In the control group, the highest basal BCKDH activity was found in the pancreas, followed by the kidney, liver, cardiac muscle, brain, skeletal muscle, and adipose tissue (Fig 2). No detectable BCKDH activity was found in the duodenal mucosa. In the skeletal muscle and adipose tissue, the high Leu diets did not stimulate BCKDH activity (Fig 2). The kidney, liver, cardiac muscle and brain showed an increase in BCKDH activity in response to feeding on the L4 diet compared to the control diet (*P* < 0.05; Fig 2). The most marked change in BCKDH activity in response to the L4 diet was found in the brain (2.5-fold), followed by the liver (1.8-fold), cardiac muscle (1.7-fold), and kidney (1.2-fold). In pigs fed the L2 diet, the BCKDH activity of these tissues showed intermediate values that were not significantly different from those of the controls. Despite the high basal BCKDH activity in the pancreas, the experimental diets had inconsistent effects on BCKDH activity (Fig 2).

### High Leu diets significantly modify the circulating BCAAs, α-keto acids, ketone bodies, and serotonin

**Fig 3:**
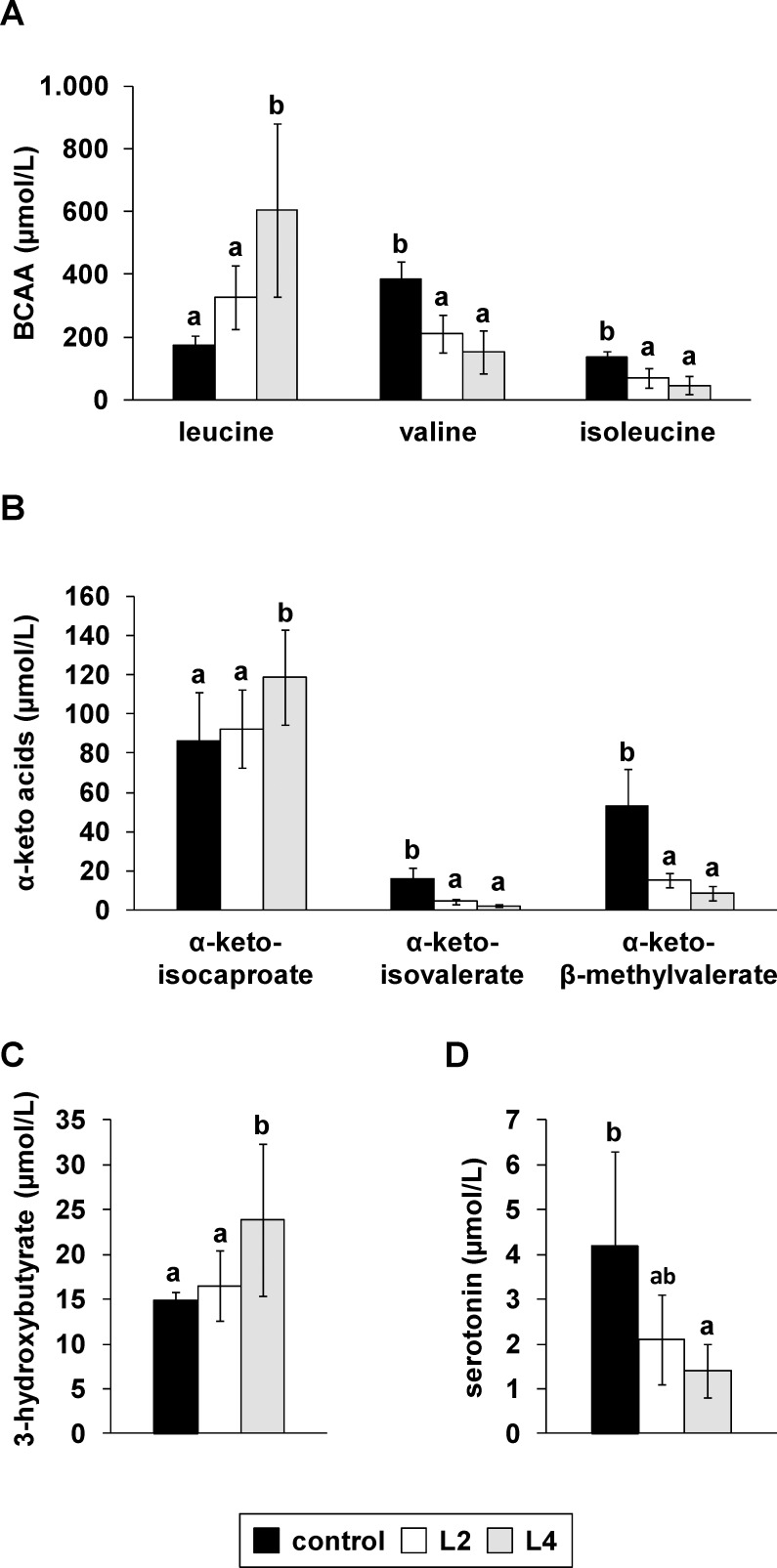
**Plasma concentrations of (A) single branched-chain amino acids, (B) keto acids, (C) 3-hydroxybutyrate and (D) serotonin of pigs in response to diets with different Leu contents.** Values represent the means ± SD of pigs fed a diet with recommended Leu content (control) or with two- (L2) and or-fold (L4) excess of Leu compared to the control diet for 35 days (n = 10). Data were analyzed by one-way ANOVA. Individual means of the treatment groups were compared by Tukey’s test if variances were homogeneous. If variances were heterogeneous, as revealed by Levene’s test, individual means were compared by Games Howell test. ^a, b^Means not sharing a common letter are significantly different from one another (*P* < 0.05).

The plasma concentration of Leu in the groups fed the L2 and L4 diets was 2.3- and 3.3-fold higher, respectively, than that in the control group, although there were strong interindividual plasma Leu responses to the high Leu diets (*P* < 0.05; Fig 3A). The increase in plasma Leu was accompanied by higher plasma levels of α-keto-isocaproate, the deamination product of Leu, and 3-hydroxybutyrate in these animals, although these alterations were significantly different between only the L4 and the control groups (*P* < 0.05; Fig 3B and 3C). Conversely, the plasma concentrations of Val and Ile and their corresponding α-keto acids α-keto-isovalerate and α-keto β-methylvalerate declined in response to the high Leu diets (Fig 3A and 3B). The plasma concentration of Trp was comparable between the three groups of pigs (S3 Table). Fig 3D demonstrates that pigs fed the high Leu diets had lower plasma concentrations of serotonin than pigs fed the control diet (P < 0.05). The ratio of BCAA:Trp in plasma was higher in the L4 group than in the control and L2 groups (control, 11.4 ± 0.8; L2, 11.1 ± 2.5; L4, 13.3 ± 2.5; *P* = 0.05).

### Excessive intake of Leu alters tissue BCAA content and cerebral Trp

**Fig 4:**
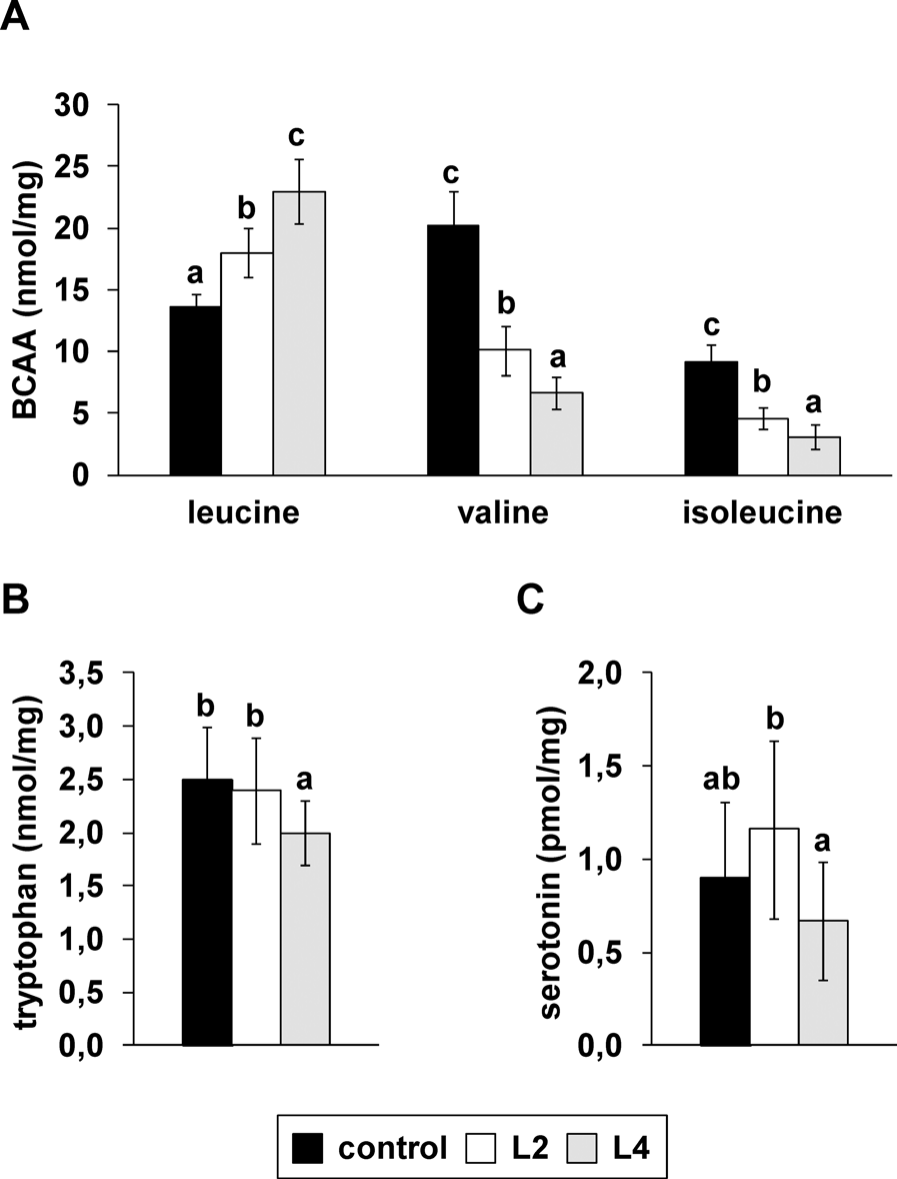
**Concentrations of (A) single branched-chain amino acids (BCAA), (B) tryptophan and (C) serotonin in the brain of pigs in response to diets with different Leu contents.** Values represent the means ± SD of pigs fed a diet with recommended Leu content (control) or with two- (L2) and or-fold (L4) excess of Leu compared to the control diet for 35 days (n = 10). Data were analyzed by one-way ANOVA. Individual means of the treatment groups were compared by Tukey’s test if variances were homogeneous. If variances were heterogeneous, as revealed by Levene’s test, individual means were compared by Games Howell test. ^a, b, c^Means not sharing a common letter are significantly different from one another (*P* < 0.05).

**Fig 5:**
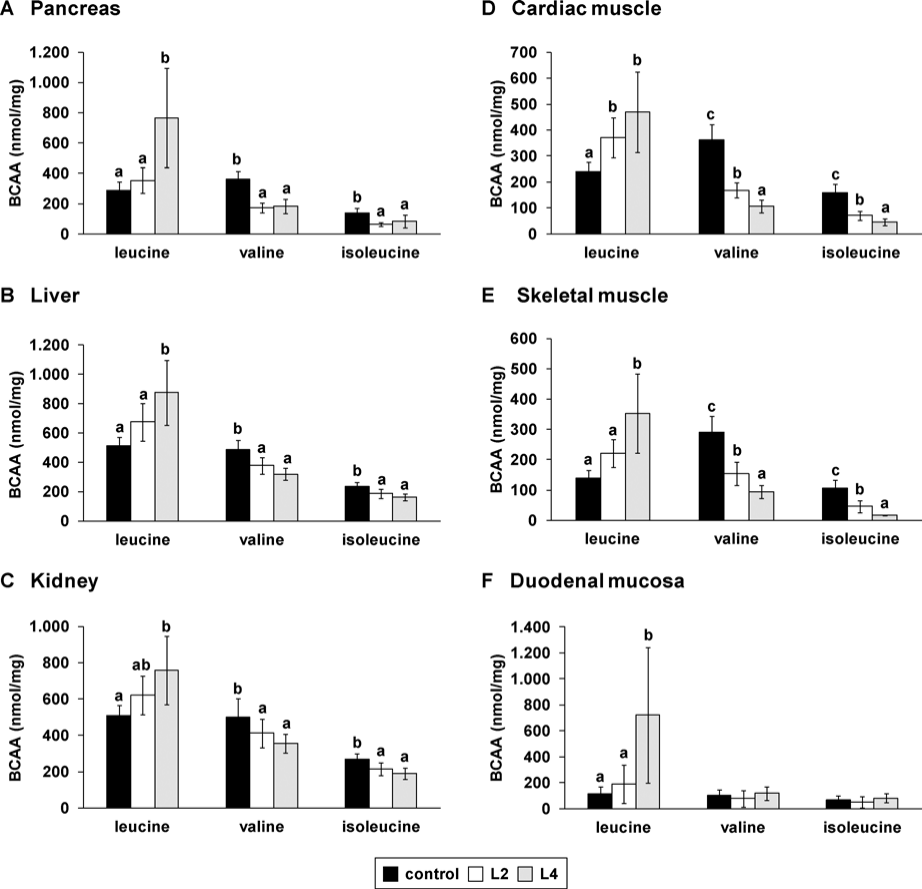
**Concentrations of (A-F) single branched-chain amino acids (BCAA) in different tissues of pigs in response to diets with different Leu contents.** Values represent the means ± SD of pigs fed a diet with recommended Leu content (control) or with two- (L2) and or-fold (L4) excess of Leu compared to the control diet for 35 days (n = 10). Data were analyzed by one-way ANOVA. Individual means of the treatment groups were compared by Tukey’s test if variances were homogeneous. If variances were heterogeneous, as revealed by Levene’s test, individual means were compared by Games Howell test. ^a, b, c^Means not sharing a common letter are significantly different from one another (*P* < 0.05).

The most marked changes of non-protein-bound amino acids in tissues in response to the high Leu diets were observed with the individual BCAAs. In line with plasma amino acid concentrations, the concentrations of Leu were markedly higher in the brain (Fig 4A, *P* < 0.05), pancreas (Fig 5A, *P* < 0.05), liver (Fig 5B, *P* < 0.05), kidney (Fig 5C, *P* < 0.05), cardiac muscle (Fig 5D, *P* < 0.05), skeletal muscle (Fig 5E, *P* < 0.05) and duodenal mucosa (Fig 5F, *P* < 0.05) of pigs fed the high Leu diets than in the controls. In particular, the Leu concentration in the duodenal mucosa was 6.2-times higher in pigs in the L4 group than in the control group. The concentrations of Val and Ile were lower in all tissues, except in the duodenal mucosa, in pigs fed the high Leu diets (Figs 4A and 5; *P* < 0.001). Other plasma and tissue amino acids showed marginal changes in response to the high Leu diets (S3–S10 Tables). Remarkably, cerebral Trp declined in response to the L4 diet (Fig 4B; *P* < 0.05). One-way ANOVA revealed a significant influence of dietary Leu on cerebral serotonin (*P* < 0.05), but the post-hoc analysis showed no significant differences in cerebral serotonin levels between the L4 and the control groups, although the L4 group had on average 26% lower serotonin than the control group (Fig 4C).

## Discussion

The current study sought to elucidate tissue-specific responses of BCKDH activity and amino acid derived metabolites in response to diets that contained 2- and 4-fold higher Leu amounts than required. We showed that pigs fed high Leu diets had lower food intake, which may explain the decline in growth observed in these animals. We further observed an increase in BCKDH activity in all tissues except the skeletal muscle and adipose tissue in response to high Leu diets. Interestingly, the highest increase in BCKDH activity in response to excessive Leu consumption was observed in the brain. The stimulated BCKDH activity and increased Leu concentration in the brain of pigs fed the high Leu diets provide evidence for elevated transport of Leu across the blood-brain barrier. Because excessive Leu in the brain is associated with neurological dysfunction [26], the observed rise in cerebral BCKDH activity is presumably a protective mechanism against Leu-associated neuronal disorders.

BCAAs and aromatic amino acids such as Trp share the same brain transporters [27,28]. Thus, we hypothesized that cerebral Trp concentration declines in response to excessive Leu consumption. Because BCAAs include not only Leu but also Ile and Val, we analyzed all three BCAAs and observed that pigs fed the high Leu diets had increased plasma concentrations of Leu but reduced concentrations of Ile and Val. The reduction of Ile and Val in response to high Leu-diets is a well-described phenomenon [8] and is caused by the stimulated BCKDH activity, which in turn degrades not only Leu but also Ile and Val. The L4 group had higher plasma BCAA levels and also a higher plasma BCAA:Trp ratio than the control and L2 groups. To investigate a possible interference between cerebral uptake of BCAAs and Trp, we analyzed a set of amino acids in the brain and found reduced concentrations of cerebral Trp in the L4 group compared to the control and the L2 group. This finding supports the hypothesis that excessive Leu consumption can hamper the cerebral uptake of Trp [29]. Trp is a precursor of serotonin. In the brain, the formation of serotonin largely depends on the availability of cerebral Trp because serotonin is not capable of crossing the blood-brain barrier [30]. ANOVA revealed a significant impact of dietary Leu on cerebral serotonin concentration, although results from the post-hoc analysis showed no differences between the high Leu groups and the control group. However, one limitation of cerebral serotonin analysis was that we quantified serotonin in a large part of the brain and not in specific cerebral regions where serotonin-synthesizing neurons are located. This limitation may have caused large standard deviations and the lack of significant differences in cerebral serotonin levels between the L4 group and the control group, although the L4 group had on average 26% lower cerebral serotonin concentration than the control group. Nonetheless, the observed changes in the composition of cerebral amino acids, in particular those of the BCAAs and Trp, are an important issue for several reasons. First, therapeutic doses of Leu used to prevent muscle atrophy are within the range used in the current study [6,7]. Second, Leu is often used in high amounts by athletes for its ability to stimulate protein synthesis in muscles. Finally, Leu supplementation is discussed as a strategy to improve performance and muscle protein synthesis in livestock production [3,4]. Although only 1% of the total serotonin in the body is synthesized in the brain, serotonin plays important roles in the brain, including control of appetite, sleep-wake rhythms, memory, temperature regulation and behavior (reviewed in [31]). Low levels of cerebral serotonin are associated with aggressive and angry behaviors, clinical depression, Parkinson’s disease, eating disorders, migraines, irritable bowel syndrome, tinnitus and bipolar diseases (reviewed in [30,32]). These possible adverse effects of high Leu diets should be considered when strategies are being developed to prevent muscle cachexia in patients and to improve the growth of livestock.

We observed lowered plasma concentration of serotonin in the pigs fed high Leu diets. Plasma serotonin is normally derived from peripheral serotonin-producing cells, and 90% of the body serotonin has been suggested to be produced by enterochromaffin cells of the gut [33]. We speculate that excessive Leu may also hamper the Trp uptake into enterochromaffin cells, thereby lowering serotonin synthesis in these cells. Considering the multiple functions of peripheral serotonin as a regulator of bone mass [5,34], platelet coagulation [35], liver regeneration [36] and its function in the gastrointestinal tract [37], the observed reduction of peripheral serotonin can potentially have further adverse effects caused by excessive Leu consumption.

Serotonin is a neurotransmitter that is involved in appetite regulation. In pigs, high consumption of Trp significantly increased food intake in these animals [38–40], which is presumably attributable to the role of Trp as a precursor for serotonin. However, on the basis of our data, we expect, that serotonin has no decisive role in reducing appetite in the L2 and the L4 groups compared to the control group. This suggestion is supported by the findings that high Leu diets had no significant effect on cerebral serotonin and that reduction in food intake was observed not only in the L4 group, but also in the L2 group, which showed no reduction in cerebral Trp compared to control pigs. We assume that appetite reduction was primarily caused by an activation of hypothalamic mTOR, which in turn stimulates anorectic signals [41,42] or by the deficiency of the indispensable amino acids such as Val and Ile that can cause an appetite reduction [43].

Leu is a ketogenic amino acid that can be degraded directly to acetyl-CoA. To test whether the high Leu diets could modify ketone body synthesis, we analyzed plasma concentration of 3-hydroxybutyrate and found that pigs in the L4 group had 60% higher levels than those in the control and L2 groups. Plasma ketone bodies are known to reduce appetite [44]. Because Km values of ketone body transporters are in the millimolar range [45], we presume that the increase in ketone bodies observed in our study does not result in a level high enough to pass the blood-brain barrier and to modulate appetite. However, it should be noted that any increase in circulating ketone bodies could contribute to the development of hyperuricemia and gout via competition for the same renal transporter as uric acid. The first step in the degradation of BCAAs is the transamination of these amino acids to form α-keto acids. This reaction is reversible and is mediated by the activity of branched-chain amino acid aminotransferase (BCAT). In this study, we observed increased plasma levels of Leu-derived α-keto-isocaproate in pigs fed the high Leu diet, which is probably caused by the stimulation of BCAT. The increase in plasma α-keto-isocaproate was accompanied by a decline of Val and Ile transamination products, namely α-keto-isovalerate and α-keto-β-methylvalerate and confirms recent findings of a study in which pigs were fed a high Leu diet [8]. In this study, we found that pigs in the L2 and the L4 groups had comparably low plasma concentrations of Val and Ile and their corresponding α-keto acids, although a tissue-specific increase in BCKDH activity was observed in the L4 group but not in the L2 group. However, in the liver, BCKDH activity showed a dose-dependent increase in response to Leu suggesting that the liver BCKDH activity, in particular, could have caused the reduction of plasma Val and Ile in the L2 pigs.

In contrast to the action of BCAT, the decarboxylation of the BCAAs by BCKDH is an irreversible step that leads to a loss of available BCAAs for protein synthesis. Thus, BCKDH activity can be used as a biomarker to assess the impact of excessive Leu supply on BCAA metabolism. We observed marked tissue-specific differences in BCKDH activity in the growing pigs with the highest basal activity in the pancreas, followed by the kidney, liver, cardiac muscle, brain, skeletal muscle and adipose tissue in pigs fed a control diet. In order to determine whether pigs could be used as a human model to study the impact of high Leu diet, we compared our data with human data. In 1998, Suryawan et al. published data on the tissue-specific BCKDH activity in humans, African green monkeys and rats and reported marked differences between their BCKDH activity patterns [10]. Although we are aware that a direct comparison between previous data [10] and our data is not possible, it is striking that the tissue-specific BCKDH activity in pigs resembles its activity in human but not rat tissues. These findings suggest that pigs could be an appropriate model to study the impact of excessive Leu consumption on metabolism.

In conclusion, excessive Leu intake can stimulate BCKDH activity in several tissues, including the brain. The changes in cerebral Trp, along with the alterations of plasma amino acid-derived metabolites such as serotonin and 3-hydroxybutyrate may limit the use of high Leu diets to treat muscle atrophy. The current findings may also be relevant to the fact that BCAAs comprise 20–40% of dietary proteins [46,47] and that protein is excessively consumed in industrialized countries [13].

## Supporting Information

S1 Table Ingredients of the experimental diets. (DOCX)

S2 Table Analyzed amino acid concentrations of the experimental diets. (DOCX)

S3 Table Effect of dietary leucine on the plasma amino acid concentrations in piglets. (DOCX)

S4 Table Effect of dietary leucine on the amino acid concentrations of pancreas in piglets. (DOCX)

S5 Table Effect of dietary leucine on the amino acid concentrations in liver of piglets. (DOCX)

S6 Table Effect of dietary leucine on the amino acid concentrations in kidney of piglets. (DOCX)

S7 Table Effect of dietary leucine on the amino acid concentrations of cardiac muscle in piglets. (DOCX)

S8 Table Effect of dietary leucine on the amino acid concentrations in skeletal muscle of piglets. (DOCX)

S9 Table Effect of dietary leucine on the amino acid concentrations of the duodenal mucosa in piglets. (DOCX)

S10 Table Effect of dietary leucine on the cerebral amino acid concentrations in piglets. (DOCX)
