# Supplementary material for: High Leucine Diets Stimulate Cerebral Branched-Chain Amino Acid Degradation and Modify Serotonin and Ketone Body Concentrations in a Pig Model
Source: PLoS One. 2016 Mar 1;11(3):e0150376. doi: 10.1371/journal.pone.0150376 (PMC4773154; doi:10.1371/journal.pone.0150376)
Supplement: S1 Table — (DOCX) [file pone.0150376.s001.docx]

**Table S1: Ingredients of the experimental diets**

| **Ingredients (%)** | **Diet** | | |
| --- | --- | --- | --- |
|  | **Control** | **L2** | **L4** |
| Wheat | 25.3 | 25.2 | 25.9 |
| Barley | 24 | 25 | 25 |
| Corn | 26.5 | 26.5 | 26.5 |
| Soybean meal | 7 | 6 | 5 |
| Whey powder | 6 | 6 | 6 |
| Wheat bran | 3 | 3 | 3 |
| Soybean oil | 2.2 | 1.8 | 1.0 |
| Monocalcium phosphate | 0.5 | 0.5 | 0.5 |
| Mineral premix^1^ | 2 | 2 | 2 |
| L-Glutamic acid | 1.6 | 1.0 | 0.0 |
| L-Histidine | 0.05 | 0.06 | 0.07 |
| L-Isoleucine | 0.14 | 0.15 | 0.17 |
| L-Leucine | 0.12 | 1.11 | 3.06 |
| L-Lysine HCL | 0.67 | 0.70 | 0.73 |
| DL-Methionine | 0.21 | 0.22 | 0.23 |
| L-Phenylalanine | 0.02 | 0.04 | 0.06 |
| L-Threonine | 0.27 | 0.28 | 0.30 |
| L-Tryptophan | 0.12 | 0.12 | 0.13 |
| L-Tyrosine | 0.05 | 0.06 | 0.07 |
| L-Valine | 0.25 | 0.26 | 0.28 |

^1^per kg premix: calcium, 265 g; sodium, 60 g; phosphorus, 40 g; magnesium, 5.5 g; iron, 2850 mg; zinc, 2200 mg; copper, 750 mg; manganese, 730 mg; cobalt, 15 mg; iodine, 10 mg; selenium, 10 mg; vitamin A, 1,000,000 IU; vitamin D, 100,000 IU; nicotinic acid, 2600 mg; vitamin E, 2000 mg; pantothenic acid, 1250 mg; riboflavin, 500 mg; pyridoxine, 300 mg; thiamin, 200 mg; vitamin K, 150 mg; folic acid, 30 mg; vitamin B12, 2 mg; biotin, 1 mg; cholin chloride, 20 g; aromatic substance, 1500 mg.
