# Supplementary material for: High Leucine Diets Stimulate Cerebral Branched-Chain Amino Acid Degradation and Modify Serotonin and Ketone Body Concentrations in a Pig Model
Source: PLoS One. 2016 Mar 1;11(3):e0150376. doi: 10.1371/journal.pone.0150376 (PMC4773154; doi:10.1371/journal.pone.0150376)
Supplement: S2 Table — (DOCX) [file pone.0150376.s002.docx]

Table S2: Analyzed amino acid concentrations of the experimental diets

| **Amino acid (%)^1^** | **Diet** | | |
| --- | --- | --- | --- |
|  | **Control** | **L2** | **L4** |
| **Leucine** | **1.09** | **1.97** | **3.75** |
| SID^2^ Leucin:Lysin | 100 | 186 | 353 |
| Histidine | 0.34 | 0.35 | 0.35 |
| Isoleucine | 0.60 | 0.60 | 0.61 |
| Lysine | 1.04 | 1.01 | 1.01 |
| Methionine+Cysteine | 0.61 | 0.61 | 0.62 |
| Phenylalanine+Tyrosine | 1.08 | 1.08 | 1.07 |
| Threonine | 0.66 | 0.66 | 0.67 |
| Tryptophan | 0.25 | 0.25 | 0.26 |
| Valine | 0.81 | 0.79 | 0.80 |

^1^analyzed in duplicate. ^2^SID = Standardized ileal digestible coefficient; SID is defined as the apparent digestibility of a nutrient corrected for the basal endogenous loss and a mean to estimate correctly the nutritional value of a nutrient. L2, pigs that received two-fold higher leucine amounts than the control; L4, pigs that received four-fold higher leucine amounts than the control.
