# Supplementary material for: High Leucine Diets Stimulate Cerebral Branched-Chain Amino Acid Degradation and Modify Serotonin and Ketone Body Concentrations in a Pig Model
Source: PLoS One. 2016 Mar 1;11(3):e0150376. doi: 10.1371/journal.pone.0150376 (PMC4773154; doi:10.1371/journal.pone.0150376)
Supplement: S3 Table — (DOCX) [file pone.0150376.s003.docx]

Table S3: Effect of dietary leucine on the plasma amino acid concentrations in piglets

| **Plasma amino acids (µmol/l)^1^** | **Diet** | | | ***P* value** |
| --- | --- | --- | --- | --- |
|  | **Control** | **L2** | **L4** |  |
| Alanine | 907 ± 138 | 823 ± 255 | 766 ± 182 | 0.330 |
| Arginine | 152 ± 50 | 142 ± 45 | 123 ± 53 | 0.448 |
| Glutamic acid | 565 ± 390 | 602 ± 449 | 725 ± 536 | 0.723 |
| Glutamine | 770 ± 96 | 695 ± 167 | 710 ± 182 | 0.521 |
| Glycine | 1135 ± 150 | 1171 ± 167 | 1203 ± 154 | 0.635 |
| Histidine | 59.1 ± 16.7 | 73.5 ± 20.3 | 80.3 ± 18.6 | 0.065 |
| Lysine | 211 ± 66 | 249 ± 125 | 214 ± 135 | 0.736 |
| Methionine | 80.5 ± 13.9 | 83.6 ± 36.6 | 92.7 ± 28.2 | 0.612 |
| Ornithine | 80.4 ± 21.0 | 90.6 ± 22.7 | 68.2 ± 22.5 | 0.105 |
| Phenylalanine | 91.7 ± 14.5 | 86.0 ± 12.3 | 78.1 ± 23.2 | 0.256 |
| Serine | 236 ± 46 | 228 ± 42 | 192 ± 21 | 0.063 |
| Threonine | 385 ± 78 | 532 ± 146 | 425 ± 167 | 0.081 |
| Tryptophan | 58.5 ± 8.0 | 56.4 ± 10.8 | 58.9 ± 22.1 | 0.936 |
| Tyrosine | 73.1 ± 11.1^ab^ | 80.9 ± 21.3^b^ | 62.0 ± 9.9^a^ | 0.056 |

^1^Data represent the means ± SD. L2, pigs that received two-fold higher leucine amounts than the control; L4, pigs that received four-fold higher leucine amounts than the control. ^a, b^Means within a row not sharing a common superscript letter are significantly different from one another (Tukey’s test; *P* < 0.05); n = 10
