# Supplementary material for: High Leucine Diets Stimulate Cerebral Branched-Chain Amino Acid Degradation and Modify Serotonin and Ketone Body Concentrations in a Pig Model
Source: PLoS One. 2016 Mar 1;11(3):e0150376. doi: 10.1371/journal.pone.0150376 (PMC4773154; doi:10.1371/journal.pone.0150376)
Supplement: S4 Table — (DOCX) [file pone.0150376.s004.docx]

Table S4: Effect of dietary leucine on the amino acid concentrations of pancreas in piglets

| **Tissue amino acids (nmol/mg)^1^** | **Diet** | | | ***P* value** |
| --- | --- | --- | --- | --- |
|  | **Control** | **L2** | **L4** |  |
| Alanine | 2575 ± 367 | 2752 ± 894 | 3046 ± 1450 | 0.635 |
| Glutamine | 1649 ± 362 | 1479 ± 492 | 1368 ± 400 | 0.355 |
| Glycine | 4147 ± 1140^a^ | 4336 ± 354^a^ | 5673 ± 1602^b^ | 0.015 |
| Histidine | 56 ± 13^a^ | 75 ± 19^a^ | 90 ± 24^b^ | 0.001 |
| Lysine | 256 ± 57 | 304 ± 118 | 274 ± 78 | 0.478 |
| Methionine | 113 ± 12 | 117 ± 28 | 130 ± 22 | 0.193 |
| Threonine | 1117 ± 361 | 1670 ± 635 | 1202 ± 844 | 0.139 |
| Tryptophan | 32 ± 6^a^ | 39 ± 7^ab^ | 50 ± 14^b^ | 0.002 |

^1^Data represent the means ± SD. L2, pigs that received two-fold higher leucine amounts than the control; L4, pigs that received four-fold higher leucine amounts than the control. ^a, b^Means within a row not sharing a common superscript letter are significantly different from one another (Tukey’s test or Games-Howell test; *P* < 0.05); n = 10
