# Supplementary material for: High Leucine Diets Stimulate Cerebral Branched-Chain Amino Acid Degradation and Modify Serotonin and Ketone Body Concentrations in a Pig Model
Source: PLoS One. 2016 Mar 1;11(3):e0150376. doi: 10.1371/journal.pone.0150376 (PMC4773154; doi:10.1371/journal.pone.0150376)
Supplement: S5 Table — (DOCX) [file pone.0150376.s005.docx]

Table S5: Effect of dietary leucine on the amino acid concentrations in liver of piglets

| **Tissue amino acids (nmol/mg)^1^** | **Diet** | | | ***P* value** |
| --- | --- | --- | --- | --- |
|  | Control | L2 | L4 |  |
| Alanine | 5060 ± 777 | 4735 ± 1122 | 3902 ± 1190 | 0.063 |
| Glutamine | 4048 ± 543 | 3901 ± 428 | 4209 ± 1322 | 0.747 |
| Glycine | 4090 ± 585^ab^ | 4188 ± 500^b^ | 3639 ± 300^a^ | 0.039 |
| Histidine | 378 ± 66 | 452 ± 84 | 480 ± 122 | 0.062 |
| Lysine | 244 ± 59 | 241 ± 46 | 204 ± 57 | 0.212 |
| Methionine | 137 ± 21 | 135 ± 19 | 137 ± 9 | 0.943 |
| Threonine | 832 ± 254^ab^ | 1105 ± 281^b^ | 767 ± 281^a^ | 0.029 |
| Tryptophan | 56 ± 7 | 58 ± 9 | 56 ± 10 | 0.830 |

^1^Data represent the means ± SD. L2, pigs that received two-fold higher leucine amounts than the control; L4, pigs that received four-fold higher leucine amounts than the control. ^a, b^Means within a row not sharing a common superscript letter are significantly different from one another (Tukey’s test or Games-Howell test; *P* < 0.05); n = 10
