# Supplementary material for: High Leucine Diets Stimulate Cerebral Branched-Chain Amino Acid Degradation and Modify Serotonin and Ketone Body Concentrations in a Pig Model
Source: PLoS One. 2016 Mar 1;11(3):e0150376. doi: 10.1371/journal.pone.0150376 (PMC4773154; doi:10.1371/journal.pone.0150376)
Supplement: S6 Table — (DOCX) [file pone.0150376.s006.docx]

Table S6: Effect of dietary leucine on the amino acid concentrations in kidney of piglets

| **Tissue amino acids (nmol/mg)^1^** | **Diet** | | | ***P* value** |
| --- | --- | --- | --- | --- |
|  | **Control** | **L2** | **L4** |  |
| Alanine | 2395 ± 234^b^ | 2053 ± 236^a^ | 1993 ± 161^a^ | 0.000 |
| Glutamine | 1056 ± 206 | 848 ± 195 | 889 ± 165 | 0.049 |
| Glycine | 8003 ± 1415^a^ | 7919 ± 300^b^ | 9486 ± 2736^a^ | 0.044 |
| Histidine | 179 ± 28 | 168 ± 28 | 172 ± 22 | 0.696 |
| Lysine | 280 ± 47 | 255 ± 51 | 236 ± 57 | 0.171 |
| Methionine | 181 ± 23 | 181 ± 32 | 175 ± 32 | 0.881 |
| Threonine | 559 ± 117 | 628 ± 210 | 554 ± 205 | 0.605 |
| Tryptophan | 88 ± 13 | 90 ± 16 | 85 ± 18 | 0.779 |

^1^Data represent the means ± SD. L2, pigs that received two-fold higher leucine amounts than the control; L4, pigs that received four-fold higher leucine amounts than the control. ^a, b^Means within a row not sharing a common superscript letter are significantly different from one another (Tukey’s test or Games-Howell test; *P* < 0.05); n = 10
