# Supplementary material for: High Leucine Diets Stimulate Cerebral Branched-Chain Amino Acid Degradation and Modify Serotonin and Ketone Body Concentrations in a Pig Model
Source: PLoS One. 2016 Mar 1;11(3):e0150376. doi: 10.1371/journal.pone.0150376 (PMC4773154; doi:10.1371/journal.pone.0150376)
Supplement: S7 Table — (DOCX) [file pone.0150376.s007.docx]

Table S7: Effect of dietary leucine on the amino acid concentrations of cardiac muscle in piglets

| **Tissue amino acids (nmol/mg)^1^** | **Diet** | | | ***P* value** |
| --- | --- | --- | --- | --- |
|  | **Control** | **L2** | **L4** |  |
| Alanine | 5169 ± 476 | 5249 ± 744 | 4901 ± 1043 | 0.593 |
| Glutamine | 9431 ± 1638^b^ | 8628 ± 1378^ab^ | 7429 ± 1764^a^ | 0.033 |
| Glycine | 1042 ± 177 | 1136 ± 133 | 1142 ± 200 | 0.361 |
| Histidine | 107 ± 19 | 123 ± 30 | 106 ± 32 | 0.315 |
| Lysine | 133 ± 40 | 129 ± 70 | 106 ± 43 | 0.494 |
| Methionine | 139 ± 33 | 152 ± 28 | 143 ± 38 | 0.648 |
| Threonine | 423 ± 168 | 651 ± 234 | 574 ± 225 | 0.066 |
| Tryptophan | 46 ± 10 | 53 ± 10 | 45 ± 14 | 0.217 |

^1^Data represent the means ± SD. L2, pigs that received two-fold higher leucine amounts than the control; L4, pigs that received four-fold higher leucine amounts than the control. ^a, b^Means within a row not sharing a common superscript letter are significantly different from one another (Tukey’s test or Games-Howell test; *P* < 0.05); n = 10
