# Supplementary material for: High Leucine Diets Stimulate Cerebral Branched-Chain Amino Acid Degradation and Modify Serotonin and Ketone Body Concentrations in a Pig Model
Source: PLoS One. 2016 Mar 1;11(3):e0150376. doi: 10.1371/journal.pone.0150376 (PMC4773154; doi:10.1371/journal.pone.0150376)
Supplement: S8 Table — (DOCX) [file pone.0150376.s008.docx]

Table S8: Effect of dietary leucine on the amino acid concentrations in skeletal muscle of piglets

| **Tissue amino acids (nmol/mg)^1^** | **Diet** | | | ***P* value** |
| --- | --- | --- | --- | --- |
|  | **Control** | **L2** | **L4** |  |
| Alanine | 1736 ± 320 | 1683 ± 310 | 1484 ± 188 | 0.148 |
| Glutamine | 2056 ± 533 | 1812 ± 220 | 1800 ± 313 | 0.261 |
| Glycine | 3330 ± 1096 | 3210 ± 682 | 2883 ± 824 | 0.467 |
| Histidine | 27 ± 20 | 45 ± 10 | 48 ± 19 | 0.073 |
| Lysine | 84 ± 31 | 93 ± 55 | 65 ± 31 | 0.327 |
| Methionine | 78 ± 14 | 85 ± 20 | 79 ± 9 | 0.538 |
| Threonine | 350 ± 103 | 491 ± 176 | 365 ± 170 | 0.096 |
| Tryptophan | 40 ± 10^b^ | 44 ± 6^b^ | 34 ± 7^a^ | 0.045 |

^1^Data represent the means ± SD. L2, pigs that received two-fold higher leucine amounts than the control; L4, pigs that received four-fold higher leucine amounts than the control. ^a, b^Means within a row not sharing a common superscript letter are significantly different from one another (Tukey’s test or Games-Howell test; *P* < 0.05); n = 10
