# Supplementary material for: High Leucine Diets Stimulate Cerebral Branched-Chain Amino Acid Degradation and Modify Serotonin and Ketone Body Concentrations in a Pig Model
Source: PLoS One. 2016 Mar 1;11(3):e0150376. doi: 10.1371/journal.pone.0150376 (PMC4773154; doi:10.1371/journal.pone.0150376)
Supplement: S9 Table — (DOCX) [file pone.0150376.s009.docx]

Table S9: Effect of dietary leucine on the amino acid concentrations of the duodenal mucosa in piglets

| **Tissue amino acids (nmol/mg)^1^** | **Diet** | | | ***P* value** |
| --- | --- | --- | --- | --- |
|  | **Control** | **L2** | **L4** |  |
| Alanine | 301 ± 95 | 262 ± 60 | 243 ± 58 | 0.261 |
| Glutamine | 130 ± 43 | 94 ± 46 | 122 ± 51 | 0.344 |
| Glycine | 223 ± 73 | 525 ± 63 | 271 ± 79 | 0.174 |
| Histidine | 18 ± 5 | 17 ± 7 | 23 ± 6 | 0.105 |
| Lysine | 93 ± 56 | 64 ± 49 | 103 ± 49 | 0.288 |
| Methionine | 55 ± 32 | 41 ± 26 | 69 ± 33 | 0.180 |
| Threonine | 100 ± 38 | 93 ± 35 | 122 ± 38 | 0.235 |
| Tryptophan | 27 ± 16 | 21 ± 12 | 34 ± 16 | 0.230 |

^1^Data are mean values ± SD. L2, pigs that received two-fold higher leucine amounts than the control; L4, pigs that received four-fold higher leucine amounts than the control.
