# Supplementary material for: High Leucine Diets Stimulate Cerebral Branched-Chain Amino Acid Degradation and Modify Serotonin and Ketone Body Concentrations in a Pig Model
Source: PLoS One. 2016 Mar 1;11(3):e0150376. doi: 10.1371/journal.pone.0150376 (PMC4773154; doi:10.1371/journal.pone.0150376)
Supplement: S10 Table — (DOCX) [file pone.0150376.s010.docx]

Table S10: Effect of dietary leucine on the cerebral amino acid concentrations in piglets

| **Tissue amino acids (nmol/mg)^1^** | **Diet** | | | ***P* value** |
| --- | --- | --- | --- | --- |
|  | **Control** | **L2** | **L4** |  |
| Alanine | 88.0 ± 9.3^b^ | 85.0 ± 7.9^ab^ | 75.5 ± 7.9^a^ | 0.036 |
| Glutamine | 603.0 ± 77.1 | 570.7 ± 75.1 | 604.7 ± 122.1 | 0.686 |
| Glycine | 120.5 ± 6.8^a^ | 114.9 ± 12.9^b^ | 134.8 ± 12.9^b^ | 0.002 |
| Histidine | 6.1 ± 0.4 | 7.3 ± 3.0 | 7.2 ± 3.3 | 0.650 |
| Lysine | 9.3 ± 1.6 | 9.3 ± 2.2 | 10.1 ± 2.7 | 0.674 |
| Methionine | 6.7 ± 0.9^b^ | 6.0 ± 1.1^ab^ | 5.0 ± 1.1^a^ | 0.004 |
| Threonine | 98.8 ± 22.0 | 122.1 ± 28.2 | 101.3 ± 32.7 | 0.163 |
| Tryptophan | 2.5 ± 0.5^b^ | 2.4 ± 0.5^b^ | 2.0 ± 0.0^a^ | 0.029 |

^1^Data represent the means ± SD. L2, pigs that received two-fold higher leucine amounts than the control; L4, pigs that received four-fold higher leucine amounts than the control. ^a, b^Means within a row not sharing a common superscript letter are significantly different from one another (Tukey’s test or Games-Howell test; *P* < 0.05); n = 10
